# A BEME (Best Evidence in Medical Education) systematic review of the use of workplace-based assessment in identifying and remediating poor performance among postgraduate medical trainees

**DOI:** 10.1186/s13643-015-0056-9

**Published:** 2015-05-08

**Authors:** Aileen Barrett, Rose Galvin, Yvonne Steinert, Albert Scherpbier, Ann O’Shaughnessy, Mary Horgan, Tanya Horsley

**Affiliations:** Education and Professional Development Unit, Royal College of Physicians of Ireland, Frederick House, 19 South Frederick St, Dublin 2, Ireland; School of Medicine, College of Medicine and Health Sciences, Brookfield Health Sciences Complex, University College Cork, Corcaigh, Ireland; Discipline of Physiotherapy, Department of Clinical Therapies, Faculty of Education and Health Sciences, University of Limerick, Limerick, Ireland; Center for Medical Education, Faculty of Medicine, McGill University, Lady Meredith House, 1110 Pine Avenue West, Montreal, Quebec H3A 1A3 Canada; Faculty of Health, Medicine and Life Sciences, University of Maastricht, Universiteitssingel 60, Maastricht, 6229 ER Netherlands; Research Unit, Royal College of Physicians and Surgeons of Canada, 774 Echo Drive, Ottawa, ON K1S 5N8 Canada; Faculty of Medicine, University of Ottawa, 451 Smyth Road, Ottawa, Ontario K1H 8M5 Canada

**Keywords:** Workplace-based assessment, Formative assessment, Postgraduate medical education, Residency training, Poor performance, Remediation, Systematic review

## Abstract

**Background:**

Workplace-based assessments were designed to facilitate observation and structure feedback on the performance of trainees in real-time clinical settings and scenarios. Research in workplace-based assessments has primarily centred on understanding psychometric qualities and performance improvement impacts of trainees generally.

An area that is far less understood is the use of workplace-based assessments for trainees who may not be performing at expected or desired standards, referred to within the literature as trainees ‘in difficulty’ or ‘underperforming’. In healthcare systems that increasingly depend on service provided by junior doctors, early detection (and remediation) of poor performance is essential. However, barriers to successful implementation of workplace-based assessments (WBAs) in this context include a misunderstanding of the use and purpose of these formative assessment tools.

This review aims to explore the impact - or effectiveness - of workplace-based assessment on the identification of poor performance and to determine those conditions that support and enable detection, *i.e.* whether by routine or targeted use where poor performance is suspected. The review also aims to explore what effect (if any) the use of WBA may have on remediation or on changing clinical practice. The personal impact of the detection of poor performance on trainees and/or trainers may also be explored.

**Methods/design:**

Using BEME (Best Evidence in Medical Education) Collaboration review guidelines, nine databases will be searched for English-language records. Studies examining interventions for workplace-based assessment either routinely or in relation to poor performance will be included. Independent agreement (kappa .80) will be achieved using a randomly selected set of records prior to commencement of screening and data extraction using a BEME coding sheet modified as applicable (Buckley *et al.*, *Med Teach* 31:282-98, 2009) as this has been used in previous WBA systematic reviews (Miller and Archer, *BMJ* doi:10.1136/bmj.c5064, 2010) allowing for more rigorous comparisons with the published literature. Educational outcomes will be evaluated using Kirkpatrick’s framework of educational outcomes using Barr’s adaptations (Barr *et al.*, *Evaluations of interprofessional education; a United Kingdom review of health and social care*, 2000) for medical education research.

**Discussion:**

Our study will contribute to an ongoing international debate regarding the applicability of workplace-based assessments as a meaningful formative assessment approach within the context of postgraduate medical education.

**Systematic review registration:**

The review has been registered by the BEME Collaboration www.bemecollaboration.org.

## Background

In 1995, Norcini *et al.* published the Mini-Clinical Evaluation Exercise, a workplace-based assessment tool specifically designed to structure feedback following an observation of a physician-patient clinical encounter [1]. Studies carried out in the late 1980s and early 1990s had articulated that doctors-in-training were very rarely provided with feedback, and even less so observed within a practice-based context [2,3]. Research was also emerging from the UK and elsewhere on ‘assessment-*for*-learning’ in which the goal of the interaction is to provide feedback on performance, inform a learning plan or action, with or without the award of a grade or mark.

Since then, over 50 tools have been developed to address specific areas of clinical practice including tools to assess clinical/procedural skills, clinical reasoning and behaviours, and there is considerable research focused on exploring the psychometric properties of the individual tools, addressing whether or not the tools used in workplace-based assessment (WBA) are valid and reliable in assessing performance [4].

A burgeoning area of interest has emerged that explores profile issues with feedback, why its impact may be limited and how trainees perceive or process that feedback [5,6]. Literature suggests that trainers feel uncomfortable giving negative feedback and structuring learning plans for trainees [5] and that trainees view WBA as merely a ‘tick-box exercise’ [6] having minimal or no impact on their perceived learning and development.

In trainees who are ‘at risk of failure’, are ‘underperforming’ or are ‘in difficulty’, left undetected this may lead to serious and, in some cases, catastrophic consequences. However, attempting to define ‘underperformance’ or ‘poor performance’ remains highly subjective in the absence of clear performance indicators. The most contemporary (2013) definition provided within a UK-based study defines the underperforming trainee as ‘requiring intervention beyond the normal level of supervisor-trainee interaction’ [7]. While this provides a descriptive definition, it does not classify the root cause of the trainee’s difficulties; rather, it provides an overarching articulation of a trainee who is not currently meeting the expectations of their training level.

Black and Welch [8] reported that of 60 doctors identified as ‘underperforming’ (in a deanery of 1482 Foundation Year 1 and 2 trainees), 16.6% of them were identified using a mini-peer assessment tool (mini-PAT) workplace-based assessment alone, while the remainder were identified by trainer observation of performance and reporting of health-related issues. In this case, formalised workplace-based assessments were no more effective than trainer observation. However, it remains unclear from the research as to whether these underperforming trainees would have been identified without any formalised WBA process.

A recent UK-based study also explored whether trainees ‘in difficulty’ use WBA differently to their peers [7]. In this setting, trainees were responsible for choosing their WBA clinical cases and assessors. Trainees who had been identified as poorly or underperforming (by other methods) did not necessarily choose less complex cases for their WBA; however, this group of trainees was more likely to approach a nursing colleague to complete a direct observation of procedural skills (DOPS) assessment and a non-clinical assessor to carry out a mini-PAT. This may, according to the authors, possibly indicate some level of avoidance of medical peers and senior colleagues among those with insight into the fact that they were underperforming. However, whether or not they approached these assessors after they had been informed they were ‘in difficulty’ is not clear.

There have been a number of published systematic reviews in the area of workplace-based assessment examining effectiveness in terms of learning or performance [4,9-12]. While the studies all cited challenges in overcoming the lack of methodological homogeneity in coming to a conclusion, the WBAs appeared to have some limited impact on performance. However, a dimension that is missing within any of these previously published systematic reviews is examining the use of WBA isolated to the context of changes from baseline for poor - or underperforming - trainees. As yet, the potential ‘ceiling effect’ of WBA rating systems is unclear; the notion that if a competence or aspect of performance is deemed to be ‘meeting’ or ‘above expectation’, a change in practice may be less likely to occur and the assessments become more of a ‘tick-box exercise’ [7]. It is therefore important to fully explore the potential of the tools to identify the poorer baseline of performance and/or to assist in improving performance from this baseline.

Our review therefore aims to further and enrich our understanding of WBA to describe and summarize how WBA affects performance, specifically among underperforming trainees. Using multiple derivatives of the concept of ‘underperforming’, we conducted an initial literature search that has identified a number of studies looking at the identification of poor performance using specific tools [8,9]; we are not yet aware however of any systematic review that has explored the use of WBA in general as a method of identifying or remediating poor performance among postgraduate medical trainees to date. Given the multiplicity of terms for describing trainees ‘in trouble’, we will use ‘underperforming’ as an umbrella term for the remainder of this review unless otherwise applicable.

## Methods/design

Using pre-established, internationally recognized, BEME (Best Evidence in Medical Education) Collaboration guidelines, we will conduct a systematic review to address the following research questions:

1. Can workplace-based assessment be used to identify and remediate underperformance among postgraduate medical trainees?
2. Of those tools thought to identify and/or remediate underperforming trainees, what features specifically contribute to their usefulness for identifying or remediating underperformance among postgraduate medical trainees?

BEME guidelines were chosen as the systematic review framework given their specificity to medical education methodology (http://www.bemecollaboration.org/Publications+Research+Methodology/). The review is not registered with the PROSPERO International Prospective Register of Systematic Reviews as the review objectives relate solely to education outcomes.

### Inclusion criteria

Only those reports that describe interventions involving the use of workplace-based assessment either routinely (*e.g.* as a component of clinical rotations) or in relation to underperformance (*e.g.* confirmation of underperformance) in postgraduate training programmes in medicine and surgery will be included. We will include studies that describe or evaluate the use of WBA within the context of the following:

- Routine or targeted use of WBA
- Trainee-led or trainer-led WBA
- Single or multiple use of WBA tools
- Use of WBAs as part of a wider programme of assessment or in the context of a range of assessment evidence
- Management or remediation of underperformance for knowledge, skills and attitudes
- Presence/absence of facilitation and/or written or verbal feedback.

No restrictions for study design will be applied; qualitative and quantitative studies will be included. However, non-research publications including commentaries, letters and editorials will not be included in the review.

### Types of outcomes

The primary outcomes of the review are those perceived to be resultant from the use of a workplace-based assessment intervention at the individual (trainee), practice (*e.g.* change from non-routine to routine use) or system-level (*e.g.* deanery-wide implementation of a new tool) changes (Table 1).

**Table 1:** **Outcomes**

**Outcome**	**Example**
Individual-level	Number of trainees identified as poorly performing through the use (either routine or targeted) of a WBA process
	Progression/remediation statistics
	Changes in trainee performance (knowledge, skills, attitudes *etc.*)
	Trainee satisfaction
Practice-level	Changes in implementation methods, *e.g.* non-routine to routine
	Implementation of new/differing WBA tools
System-level	Changes in system-wide implementation of WBA tools or methods, *e.g.* throughout a deanery

Secondary outcomes will include the conditions under which the use of WBA is most useful in identifying or remediating underperformance and, where possible, the features of WBA tools, or factors in using WBA, that are most likely to contribute to successful remediation of underperformance. Educational outcomes will be evaluated using Kirkpatrick’s framework of educational outcomes using Barr’s adaptations for medical education research [13].

### Search strategy and sources

Search strings will be iteratively developed between project, content and information scientist expertise using a dynamic combination of MeSH (medical subject headings) and free-text terms to ensure breadth and depth of coverage. Once the search has been tested and validated for optimal precision and recall, all electronic databases (see below) will be searched to identify potentially relevant records using appropriate derivatives of the searches with a search adapted as needed. Prior to final searching, we anticipate the MEDLINE search to be peer reviewed using the PRESS (Peer Review of Search Strategies) model.

Given the known complexity of searching and disparate indexing practices of medical education literature [14] and to ensure comprehensiveness of our search, the following electronic databases will be searched: MEDLINE, CINAHL, British Education Index, EMBASE, ERIC, Australian Education Index, BEME published reviews, Cochrane, DARE, PsycINFO and Science Direct. Our searches will be limited to 1995 to the most recent search date. Only English-, French-, German- and Dutch-language reports will be considered for inclusion and were chosen to reflect the abilities of the review authors.

The complexity of searching and variability with terminology within the field of workplace-based assessments will also be addressed; to ensure comprehensiveness and reduce the likelihood of missing relevant research we will supplement searches by reviewing the reference lists of included studies and review articles [15]. Given the productivity of research in workplace-based assessment, our team will conduct a validity check through contact with prominent authors in the field of workplace-based assessment for expert recommendations and guidance and to identify unpublished (including doctoral theses), recently published or ongoing studies relevant to this review to ensure missed or ongoing research is identified and included.

### Data collection and analysis

#### Study selection

Titles and abstracts of records will be reviewed in duplicate using a well-accepted algorithm that sees only one review for studies thought to be ‘included’ and two independent assessments for those thought to be ‘excluded’ at title and abstract screening. Full texts of the potentially relevant articles will be reviewed in duplicate to determine inclusion using pre-defined assessment criterion; conflicts will be resolved as needed.

#### Data extraction and management

Using a BEME coding sheet modified to suit specific review needs, two study authors (AB and RG) will independently extract data from all relevant studies. Prior to full extraction, the two authors will engage in a process of orientation to the tool *a priori* to full extraction to ensure inter-rater reliability to a kappa of 0.80 agreement. Conflicts will be resolved as needed and a third assessor will be consulted to assess validity/accuracy of responses as needed (TH, YS, AS).

#### Methodological quality

Internal validity of each study will be evaluated using the BEME criteria as this has been used in previous WBA systematic reviews [9] allowing for more meaningful comparisons with the published literature. Recognizing limitations around reporting quality, we will include a formal risk of bias assessment for any identified randomized trials [16] and observational studies [17]; the COREQ (consolidated criteria for reporting qualitative research) will be used to evaluate the quality of any qualitative studies included [18]. We propose to modify one of the BEME quality criteria (‘control for confounding’) to include author ‘positionality’ and risk of bias assessment (Table 2), key features of constructivist and, to some extent, post-positivist research methodologies including grounded theory [19]. Many studies lack an exploration or explicit declaration of the author’s ‘position’ within or outside the research, a feature which may assist in determining the quality of the published research [20,21].

**Table 2:** **BEME quality indicators (Buckley** ***et al.*** [24]**)**

	**Questions**
Research question	Is the research question or hypothesis clearly stated?
Study subjects	Is the subject group appropriate for the study being carried out?
Data collection methods	Are the methods used appropriate for the research question and context?
Completeness of data	Attrition rates/acceptable questionnaire response rates
Risk of bias assessment	Is a statement of author positionality and a risk of bias assessment included?
Analysis of results	Are the statistical and other methods of results analysis used appropriate?
Conclusions	Is it clear that the data justify the conclusions drawn?
Reproducibility	Could the study be repeated by other researchers?
Prospective	Is the study prospective?
Ethical issues	Are all ethical issues articulated and managed appropriately?
Triangulation	Were results supported by data from more than one source?

### Synthesis of extracted evidence

Study data will be analysed and classified according to the primary and secondary outcomes identified.

Based on our literature search to date and the consistent conclusions of the systematic reviews discussed earlier, one of the most significant challenges in appraising WBA literature is the lack of homogeneity between study methods. We anticipate that heterogeneity may be present within our subset of literature and thus meta-analysis is unlikely.

However, the team plans to explore and quantify heterogeneity of quantitative studies using a standard test of heterogeneity (*e.g. I*^2^) and visually using funnel plots to identify and explore outliers. Descriptive synthesis, as described by Saedon *et al.* [11], will also be considered. In the event that heterogeneity of studies precludes quantitative syntheses (*e.g.* extensive subject or statistical heterogeneity), a rich descriptive synthesis including post hoc, exploratory work that attempts to explain differences in findings [22] will be undertaken.

In the case of qualitative studies included for analysis, we will use a qualitative meta-synthesis analysis method to explore the common themes and concepts [23] emerging from the research studies.

## Discussion

The findings of this review will have important implications for the use of workplace-based assessment internationally particularly regarding advancing the science of workplace-based assessments within the context of trainees in difficulty. The early identification of underperformance remains a challenge for medical educators, and this review will explore the role, if any, of WBA in that early identification and remediation.

## Abbreviations

DOPS: Direct observation of procedural skills
Mini-CEX: Mini-clinical evaluation exercise
Mini-PAT: Mini-peer assessment tool
WBA: Workplace-based assessment
